# Interface-engineered ferroelectricity of epitaxial Hf_0.5_Zr_0.5_O_2_ thin films

**DOI:** 10.1038/s41467-023-37560-3

**Published:** 2023-03-30

**Authors:** Shu Shi, Haolong Xi, Tengfei Cao, Weinan Lin, Zhongran Liu, Jiangzhen Niu, Da Lan, Chenghang Zhou, Jing Cao, Hanxin Su, Tieyang Zhao, Ping Yang, Yao Zhu, Xiaobing Yan, Evgeny Y. Tsymbal, He Tian, Jingsheng Chen

**Affiliations:** 1 Department of Materials Science and Engineering, National University of Singapore, 117575 Singapore, Singapore; 2 School of Materials and Energy, Electron Microscopy Centre of Lanzhou University and Key Laboratory of Magnetism and Magnetic Materials of the Ministry of Education, Lanzhou University, Lanzhou, 730000 PR China; 3 Center of Electron Microscope, State Key Laboratory of Silicon Materials, School of Materials Science and Engineering, Zhejiang University, Hangzhou, 310027 China; 4 Department of Physics and Astronomy and Nebraska Center for Materials and Nanoscience, University of Nebraska, Lincoln, NE 68588-0299 USA; 5 Department of physics, Xiamen University, Xiamen, 361005 China; 6 Key Laboratory of Brain-Like Neuromorphic Devices and Systems of Hebei Province, Hebei University, Baoding, 071002 PR China; 7 Institute of Materials Research and Engineering, Agency for Science, Technology and Research (A*STAR), 138634 Singapore, Singapore; 8 Singapore Synchrotron Light Source (SSLS), National University of Singapore, 5 Research Link, 117603 Singapore, Singapore; 9 Institute of Microelectronics, Agency for Science, Technology and Research (A*STAR), 138634 Singapore, Singapore; 10 School of Physics and Microelectronics, Zhengzhou University, Zhengzhou, 450052 China

**Keywords:** Ferroelectrics and multiferroics, Surfaces, interfaces and thin films

## Abstract

Ferroelectric hafnia-based thin films have attracted intense attention due to their compatibility with complementary metal-oxide-semiconductor technology. However, the ferroelectric orthorhombic phase is thermodynamically metastable. Various efforts have been made to stabilize the ferroelectric orthorhombic phase of hafnia-based films such as controlling the growth kinetics and mechanical confinement. Here, we demonstrate a key interface engineering strategy to stabilize and enhance the ferroelectric orthorhombic phase of the Hf_0.5_Zr_0.5_O_2_ thin film by deliberately controlling the termination of the bottom La_0.67_Sr_0.33_MnO_3_ layer. We find that the Hf_0.5_Zr_0.5_O_2_ films on the MnO_2_-terminated La_0.67_Sr_0.33_MnO_3_ have more ferroelectric orthorhombic phase than those on the LaSrO-terminated La_0.67_Sr_0.33_MnO_3_, while with no wake-up effect. Even though the Hf_0.5_Zr_0.5_O_2_ thickness is as thin as 1.5 nm, the clear ferroelectric orthorhombic (111) orientation is observed on the MnO_2_ termination. Our transmission electron microscopy characterization and theoretical modelling reveal that reconstruction at the Hf_0.5_Zr_0.5_O_2_/ La_0.67_Sr_0.33_MnO_3_ interface and hole doping of the Hf_0.5_Zr_0.5_O_2_ layer resulting from the MnO_2_ interface termination are responsible for the stabilization of the metastable ferroelectric phase of Hf_0.5_Zr_0.5_O_2_. We anticipate that these results will inspire further studies of interface-engineered hafnia-based systems.

## Abstract (web summary)

Ferroelectric hafnia-based thin films are promising for applications in memories and neuromorphic devices due to their robust ferroelectricity at reduced dimensions. Here, the authors demonstrate stabilization of the metastable orthorhombic phase in Hf_0.5_ Zr_0.5_O_2_ films by interface engineering with a hole doping mechanism.

## Introduction

The discovery of ferroelectricity in Si-doped HfO_2_ thin films in 2011^1^ has triggered extensive research and technological interest in this material. Due to robust ferroelectricity at reduced dimensions^2–4^ and compatibility with complementary metal-oxide-semiconductor (CMOS) technology, these films are promising for applications in ferroelectric-random access memories (FeRAMs), ferroelectric-field effect transistors (FeFET), and neuromorphic devices^5–14^. At high temperatures, HfO_2_ possesses a high-symmetry cubic phase (${{{{{\rm{F}}}}}}m\bar{3}m$), which transforms into the tetragonal phase ($P{4}_{2}/{nmc}$) at 2773 K and then to the monoclinic phase ($P{2}_{1}/c$) at 1937 K upon cooling^15^. However, the crystal structures of these polymorphs of HfO_2_ are non-ferroelectric. It has been recognized that the ferroelectricity in HfO_2_-based materials is related to the low symmetry orthorhombic phase (${Pca}{2}_{1}$)^16^, which can be stabilized with the help of thin-films growth^17–20^. Therefore, the rational engineering capability of the thin-film growth becomes critical for achieving robust ferroelectricity in HfO_2_-based materials.

Hf_0.5_Zr_0.5_O_2_ (HZO) has the most robust ferroelectric properties among the reported doped-hafnium oxides^4,21–32^. However, up to date, most of the HZO thin films studied were polycrystalline, which contained secondary non-ferroelectric phases that suppressed the ferroelectric properties and obscured the understanding of the fundamental physics of ferroelectric HZO^17,33,34^. It was demonstrated that the ferroelectric orthorhombic phase can be stabilized in epitaxial HZO films grown on a perovskite substrate^35–41^. Notably, it was found that the La_0.67_Sr_0.33_MnO_3_ (LSMO) buffer layer is essential in the epitaxial growth of ferroelectric HZO^18,19,36,42^. Given that fluorite-like HZO and perovskite LSMO have different structures, there is a lattice mismatch between them. A few mechanisms for obtaining the ferroelectric phase of HZO by the LSMO buffer layer have been proposed, such as the interface reconstruction^18^ and the domain matching epitaxy^43^. Recently, ref. ^44^ reported that chemical reconstruction at the HZO/LSMO interface, where the substitution of the Mn cations by Hf or Zr cations occurs during the epitaxial growth of HZO on LSMO. However, the role of LSMO in the stabilization of the ferroelectric *o*-phase is still unclear, and its physical mechanism is not well understood.

In this work, we demonstrate the enhanced stabilization of the ferroelectric *o*-phase in HZO by the deliberate control of the termination of the bottom LSMO layer, namely, the MnO_2_ termination or the LaSrO termination. We find that HZO films grown on the MnO_2_-terminated LSMO exhibit a more enhanced ferroelectric phase than those grown on the LaSrO-terminated LSMO. We provide a useful strategy to stabilize the ferroelectric phase in ultrathin HZO films^45,46^. Even though the HZO layer is as thin as 1.5 nm, the clear ferroelectric orthorhombic (111) crystal orientation is observed on the MnO_2_-terminated LSMO. Based on scanning transmission electron microscopy (STEM) measurements and density functional theory (DFT) calculations, we argue that hole doping of HZO resulting from the electrostatic potential difference at the interface is the origin of the enhanced fraction of the ferroelectric *o*-phase of HZO.

## Results

### Interface engineering of epitaxial HZO films

**Fig. 1:**
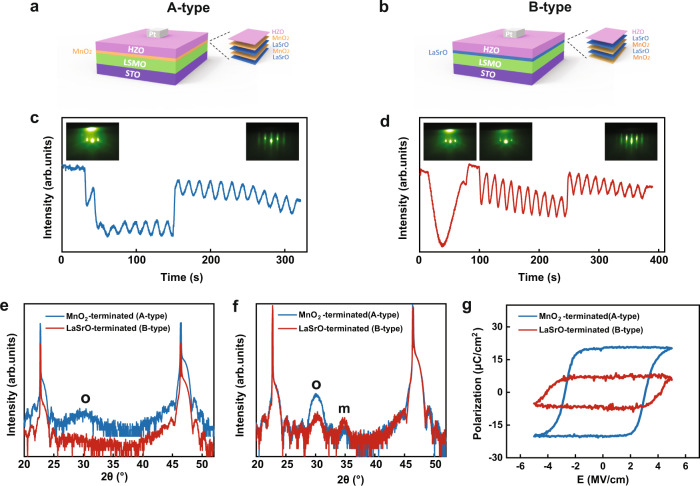
RHEED, XRD characterization, and ferroelectric characterization of A- and B-type HZO/LSMO heterostructures. **a**, **b** Schematic of the A-type (HZO/MnO_2_) terminated and B-type (HZO/LaSrO) terminated structure. The atomic plane sequences across the HZO/LSMO interfaces are indicated. **c**, **d** RHEED intensity oscillations of the specularly reflected beam during the growth of A-and B-type structure. The insets indicate the RHEED patterns before and after the growth of each layer. **e**, **f** *ω−2θ* scan of XRD characterization for A-type and B-type heterostructures with HZO thickness of (**e**) 1.5 nm and (**f**) 8 nm. “o” indicates the orthorhombic phase HZO and “m” indicates the monoclinic phase HZO. **g** Positive-up-negative-down (PUND) polarization-electric field loops for the A-type (blue) and B-type (red) heterostructures. The HZO layer thickness is *t*_HZO_ = 8 nm.

We utilize the state-of-the-art growth technique^47^ to rationally fabricate two types of HZO/LSMO structures, which are different by the interface termination of LSMO: one has the MnO_2_ termination (A-type) and the other has the LaSrO termination (B-type), as schematically shown in Fig. 1a, b. The HZO/MnO_2_ terminated structure is obtained by growing LSMO layer-by-layer on top of the treated SrTiO_3_ substrate with TiO_2_ termination, while the HZO/LaSrO-terminated structure is obtained by inserting a SrRuO_3_ layer between LSMO and treated SrTiO_3_ substrate, where the volatile RuO_2_ is evaporated at the elevated temperature and SrO termination is remained (see the “Methods” section for the detailed growth strategy). The film thickness and the interfacial termination are precisely controlled with the help of reflection high-energy electron diffraction (RHEED) monitoring (Fig. 1c, d). The x-ray reflectivity measurements, as shown in Supplementary Fig. S1, further confirm the smooth interface and the thickness of HZO films. Figure 1e, f show the crystallographic information of these two types of heterostructures (with HZO thickness of 1.5 and 8 nm, respectively) obtained by XRD measurements. It is seen that in both structures, a characteristic peak at about 30° is observed at the HZO thickness of 8 nm, which represents the orthorhombic phase HZO (*o*-HZO (111)). Another characteristic peak located at about 35° in the measured XRD data represents the monoclinic phase of HZO (*m*-HZO (002)). The coexistence of the *o*- and *m*-phases is common for the epitaxial HZO films, and generally, the fraction ratio of the *o*-phase to *m*-phase decreases with the increase of the HZO film thickness^35^. Notably, we find that the XRD peak of ferroelectric *o*-phase HZO in the A-type heterostructure is stronger than that in the B-type heterostructure, while the peak of non-ferroelectric *m*-phase HZO is less pronounced, indicating that the A-type heterostructure contains a greater fraction of the ferroelectric *o*-phase HZO component. Moreover, for an HZO thickness of 1.5 nm, *o*-HZO (111) can only be observed on the HZO/MnO_2_ terminated sample. This is consistent with the previous report, where the *o*-HZO (111) phase was also not formed on the mixed termination of the LSMO buffer layer (untreated LSMO)^18^. This result indicates that with the MnO_2_ terminated buffer layer, the fundamental limits of ferroelectric HZO *o*-phase can be enhanced and pushed to below 1.5 nm. The enhancement of the ferroelectric *o*-phase on MnO_2_ termination is consistently observed when the HZO layer thicknesses are 3 and 5 nm (Supplementary Fig. S2).

### Ferroelectric properties of HZO films with different terminations of LSMO

Ferroelectric polarization-electric field loops (P-E loops) for A- and B-type heterostructures allow characterizing the ferroelectric polarization quantitatively. These measurements are performed using HZO films with deposited Pt top electrode films. As seen from Fig. 1g, the A-type heterostructure (with an HZO thickness of 8 nm) has a higher remnant polarization value than that of the B-type heterostructure. This result is in line with our XRD and STEM characterizations, suggesting that the A-type heterostructure favors the formation of ferroelectric *o*-phase HZO and therefore yields a higher *P*_r_. Additionally, to further examine the ferroelectricity in our ultrathin HZO film, the in-plane P-E loop measurement for the A-type heterostructure sample (with an HZO thickness of 1.5 nm) is performed (Supplementary Fig. S3). The bistable current-voltage loops (Supplementary Fig. S4) show clear switching current and non-leaky characteristics of our heterostructures. It is interesting to note that the A-type heterostructure shows steady P-E loops upon cycling (Supplementary Fig. S5a), without the widely observed wake-up cycling required for other hafnia-based systems^21^, while the B-type heterostructure shows obvious wake-up behavior in the first 1000 cycles (Supplementary Fig. S5b). Generally, the diffusion and redistribution of oxygen vacancies is believed to account for the wake-up effect^21,48–51^. Our results suggest that the wake-up may be attributed to the existence of the non-ferroelectric phase in the films. The interfacial engineering strategy can effectively stabilize the ferroelectric *o*-phase and thus avoid the wake-up behavior in HZO film. In addition, we performed an endurance test for the HZO films, and remarkably, the sample with A-type heterostructure did not break down after 10^9^ cycles using the same poling voltage (Supplementary Fig. S5c). Our result shows a high-level endurance of the epitaxial HZO films grown by the interface engineering strategy^52,53^. Lyu et al.^54^ reported an endurance of over 10^11^ cycles for epitaxial HZO films with a wake-up effect. For those epitaxial HZO films without wake-up cycling, the endurance is generally lower than 10^6^ (ref. ^18^).

The ferroelectric switching behavior of the HZO films with two heterostructures are studied by the piezo-response force microscopy (PFM) technique. The ferroelectric domain switching of the HZO films with a film thickness of 8 and 1.5 nm is demonstrated by applying the positive and negative poling voltages to them (see Supplementary Figs. S6–8). Our result shows good retention of ferroelectric switching of 8 nm HZO samples with two types of heterostructures (Supplementary Fig. S7b, f). The retention of ferroelectric switching of A-type heterostructure with an HZO thickness of 1.5 nm is shown in Supplementary Fig. S8e. However, a clear ferroelectric switching PFM contrast is very difficult to obtain in the B-type heterostructure with an HZO thickness of 1.5 nm (Supplementary Fig. S8c), which is in line with our XRD data (Fig. 1e) that no ferroelectric *o*-phase is observed in this sample.

### Stabilization of ferroelectric *o*-phase and interface reconstruction

**Fig. 2:**
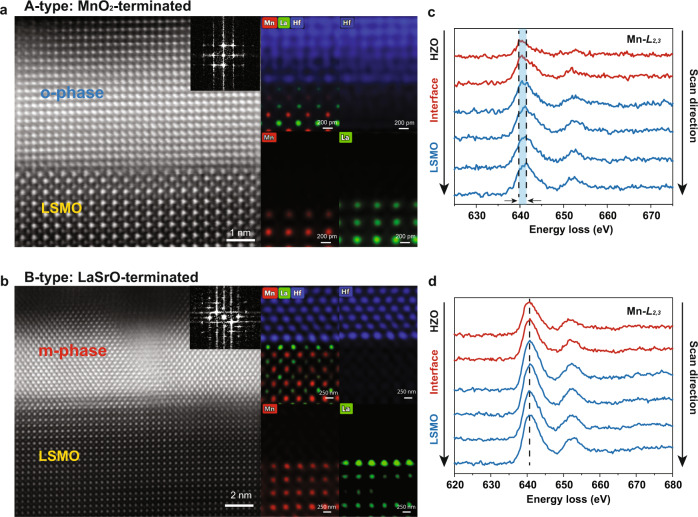
HAADF-STEM characterization. **a** Cross-section image and atomically resolved EDX for A-type heterostructure. **b** Cross-section image and atomically resolved EDX for B-type heterostructure, which are observed along the [110] zone of the substrate. Mn, La, and Hf atoms in the EDX maps are seen in red, green, and blue colors, respectively. **c**, **d** Layer-resolved EELS spectra of Mn-L_2,3_ edge for **c** A-type and **d** B-type heterostructures. Arrows indicate the scan direction. Dashed lines mark the Mn-L_2,3_ peak positions. The blue area between the dashed lines indicates the energy loss shift of the Mn-L edge.

**Fig. 3:**
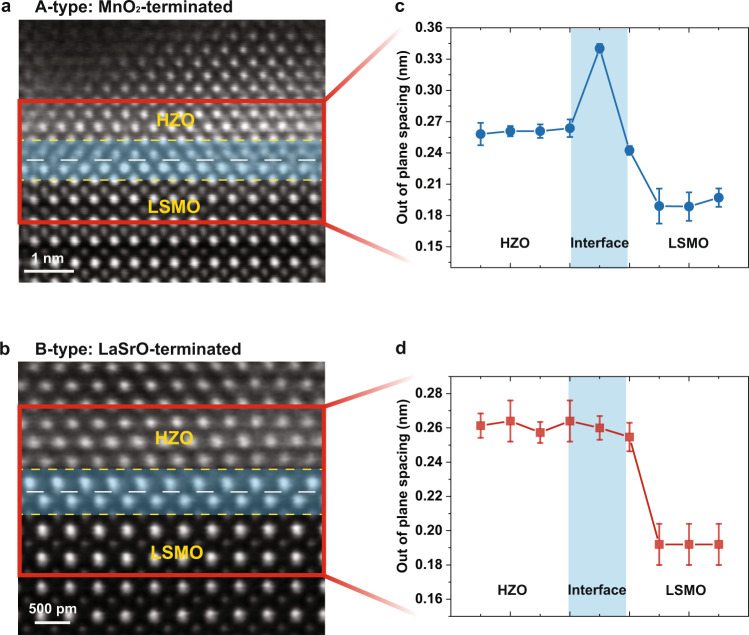
HAADF-STEM images and out-of-plane analyzation. **a**, **b** Cross-section images of A- and B-type heterostructures. The white dashed lines indicate the interfaces between HZO and LSMO. **c**, **d** Out-of-plane spacing within the red-frame region in A- and B-type heterostructure samples. The regions confined by the yellow dotted lines in (**a**, **b**) correspond to the blue color regions in (**c**, **d**). Error bars are calculated from the out-of-plane measurements of different regions for each sample.

STEM and electron energy-loss spectroscopy (EELS) characterization are performed to further provide insights into the structural properties of the two heterostructures. Figure 2a, b show high-angle annular dark field (HAADF) images and atomic-resolution energy-dispersive x-ray (EDX) elemental maps of the cross-section of the A- and B-type heterostructures. The bottom LSMO layer has a perfect epitaxial (001) relationship with a slightly compressed c-axis with respect to the STO substrate. The phase identification in the two heterostructures is performed by analyzing the atomic structure in HAADF-STEM images and combining with Fast Fourier Transform (FFT) (Supplementary Fig. S9). At the same time, the HZO demonstrates a highly textured growth on the bottom LSMO layer. We find that in the A-type heterostructure, the majority phase of HZO is the ferroelectric *o*-phase, while the minority phase is the non-ferroelectric *m*-phase (Fig. 2a, also see Supplementary Fig. S10). On the contrary, in the B-type heterostructure, the *m*-phase has a larger fraction compared to the A-type heterostructure (Fig. 2b). Statistics analyzation of *m*-phase and *o*-phase crystalline grains helps to quantify the *m*-phase and the *o*-phase distribution in two heterostructures (see Supplementary Fig. S11). This microscopic observation by STEM is consistent with the macroscopic XRD results of the two types of structures. However, as is evident from the EDX elemental maps (Fig. 2a, b, right panels), the terminations of the LSMO layers in the A- and B-type heterostructures appear to be similar, both exhibiting a stronger LaSrO EDX contrast at the atomic plane terminating LSMO. This is unexpected for the precise layer-by-layer growth mode designed for the two types of structures. The difference between the A- and B-type interface structures occurs in the HZO layer regions adjacent to the interface. Our out-of-plane spacing analysis (Fig. 3) provides evidence for the interface reconstruction in the A-type heterostructures. Figure 3c shows the spacing between atomic layers for the A-type sample. It is seen that the HZO atomic layer spacing at the interface region is about 0.34 nm, which is much larger than that away from the interface (about 0.26 nm). For the B-type sample, the HZO atomic layer spacing at the interface is about 0.26 nm (Fig. 3d), basically the same as the spacing at the *m*-HZO/LSMO interface region. This difference indicates that an interface reconstruction occurs in the A-type HZO/LSMO heterostructure. A similar result was obtained by Estandía et al.^42^, who argued that the HZO/LSMO interface reconstruction is associated with partial substitution of the interfacial Mn atoms with Hf (Zr). This result can be understood in terms of the polar nature of the pristine MnO_2_-terminated LSMO surface and the nominal ionic charges of Hf and Mn cations. Due to a higher ionic charge of Hf^+4^ compared to Mn^+3.33^ in LSMO, the substitution of Hf for Mn is energetically favorable due to the reduced polarity of the interface^42^. A similar phenomenon was also reported for LaFeO_3_/n-SrTiO_3_ heterostructures^55^.

### Charge transfer at the LSMO/HZO interface

In addition to the interface reconstruction, we observe an effect of charge transfer at the LSMO/HZO interface evident from our atomically resolved EELS data (Fig. 2c, d). For the A-type heterostructure, we detect an energy loss shift of about 1.25 eV of the Mn-L edge from the bottom to the top across the interface (Fig. 2c), indicating a decrease in the Mn valence^56^. In contrast, no such energy shift of the Mn-L edge is observed for the B-type heterostructure (Fig. 2d). Combined with the structural characterization results (Fig. 1), our EELS analysis suggests an important role of the charge transfer in the stabilization of the ferroelectric phase in HZO thin films^57^.

**Fig. 4:**
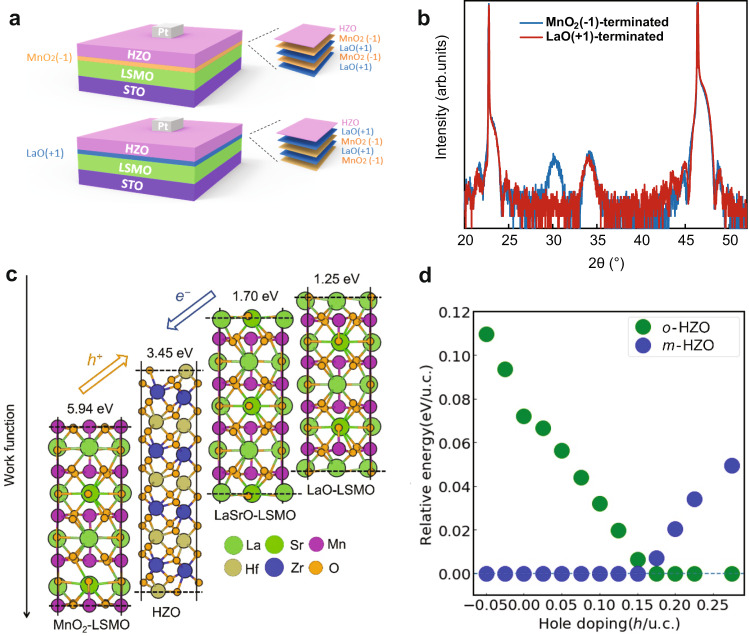
Interface charge tuning and theoretical results. **a** Schematic of the MnO_2_^(−1)^-terminated structure (top) and LaO^(+1)^-terminated structure (bottom) obtained by inserting a 2-unit-cell LaMnO_3_ layer between HZO and LSMO layers. The resulting termination is MnO_2_^(−1)^ with a negative ionic charge or LaO^(+1)^ with a positive ionic charge for A- and B-type heterostructures, respectively. **b** XRD data for the MnO_2_^(−1)^-terminated structure (blue curve) and the LaO^(+1)^-terminated structure (red curve). **c** Calculated work functions of *o*-HZO, MnO_2_-terminated LSMO (MnO_2_-LSMO), LaSrO-terminated LSMO (LaSrO-LSMO), and LaO-terminated LSMO (LaO-LSMO). MnO_2_-terminated LSMO donates holes to HZO, while LaSrO- and LaO-terminated LSMO donates electrons. **d** Relative stability of orthorhombic (green circles) and monoclinic (blue circles) HfO_2_ phases as a function of hole doping density. The lowest energy phase is used as the reference, and its energy is set to zero. The energy of the other phase is given with respect to the most stable one.

To further support this inference, we fabricated two control samples with a 2-unit-cell LaMnO_3_ layer inserted between HZO and LSMO layers in both A- and B-type heterostructures (see “Methods”). The motivation for this test was the control of the interface ionic charge and, thus, interface charge transfer between the LSMO and HZO layers in the heterostructures. A pristine La_0.67_Sr_0.33_MnO_3_ (001) surface with MnO_2_-termination has a net charge of −0.67, while that surface with the LaSrO-termination has a charge of +0.67. On the contrary, the structure with the inserted LaMnO_3_ leads to MnO_2_^(−1)^-termination with a negative charge and LaO^(+1)^-termination with a positive charge (Fig. 4a), for A- and B-type heterostructures, respectively. We found that the MnO_2_^(−1)^-terminated structure shows strong peaks of both *o*-phase and *m*-phase HZO, while the LaO^(+1)^-terminated structure only shows a strong peak of *m*-phase HZO (Fig. 4b). This observation indicates that a negative ionic charge of the MnO_2_-termination plays a key role in the formation of *o*-phase in the HZO film, while a positive charge of the LaO-termination may hinder the formation of the *o*-phase in HZO or facilitate the *m*-phase in HZO. Our control experiment strongly supports the important role of the charge transfer at the LSMO/HZO interface in the stabilization of the *o*-phase in the HZO films.

### Hole doping mechanism for stabilization of ferroelectric *o*-phase

The importance of the charge transfer in the stabilization of the ferroelectric *o*-phase is confirmed by our DFT calculations (see “Methods” for details). The appearance of the charge transfer is qualitatively inferred from the comparison of the calculated work functions of HZO and LSMO with A- and B-type surface terminations. As shown in Fig. 4c, the work function of *o*-HZO (defined as the energy difference between the vacuum level and the valence band maximum) is calculated to be 3.45 eV. The work functions of the LaO-, LaSrO-, and MnO_2_-terminated LSMO are calculated to be 1.25, 1.70, and 5.94 eV, respectively. These differences in the work functions demonstrate that during the epitaxial growth of *o*-HZO films on LSMO, there is charge transfer between the *o*-HZO and LSMO layers. Due to the larger work function of the MnO_2_-terminated LSMO (5.94 eV) compared to *o*-HZO (3.45 eV), the MnO_2_-terminated LSMO is expected to attract electrons from HZO and become negatively charged during epitaxial growth of HZO. This is consistent with our experimental observations of the reduced valence of the Mn ions at the MnO_2_-terminated interface. Therefore, HZO films grown on MnO_2_-terminated LSMO are under electron-deficient (or hole-rich) conditions. These conditions are supportive of the ferroelectric *o*-phase of HZO, as found in our experiments. In contrast, HZO films grown on LaO- and LaSrO- terminated LSMO are under electron-rich conditions, where the non-ferroelectric *m*-phase is the dominant structural phase of HZO detected in our experiments. To further prove that hole doping could stabilize the ferroelectric phase of HZO, we calculate the energy difference between *o*-HZO and *m*-HZO phases for different levels of doping. The results are displayed in Fig. 4d. It is seen that the *m*-phase of HZO is energetically more favorable than the *o*-phase at zero doping. Hole doping gradually reduces the energy difference between *o*-HZO and *m*-HZO phases. Therefore, hole doping is favorable for stabilizing the *o*-HZO phase. When the hole concentration becomes larger than 0.17 h/u.c., the *o*-HZO phase becomes the ground state. Electron doping, on the other hand, is detrimental to the stabilization of the *o*-HZO phase.

## Discussion

All the above results clearly show that extra holes contribute to stabilizing the ferroelectric phase of HZO, and the stabilization effect is determined by the amount of charge exchange between the HZO film and the substrate. This is also consistent with our experimental observations on different substrates. During the synthesis process, we adopted both LSMO and LMO buffer layers to achieve the ferroelectric phase of HZO films. For both LaO-terminated LMO and LSMO (or LaO-termination), we can only grow the nonpolar *m*-HZO phase, because LaO-termination donates electrons to HZO, which can only facilitate the growth of *m*-HZO (Fig. 4b). For MnO_2_-terminated substrates; however, there are differences in final growth phases of HZO films. We achieve almost pure *o*-HZO films on the MnO_2_- terminated LSMO, but we can only synthesize mixed *o*- and *m*-HZO phases on the MnO_2_-terminated LMO. These differences could also be understood from surface work functions and involved charge exchanges during the synthesis process. For MnO_2_-terminated LSMO and LMO, their surface work functions are calculated to be 5.94 and 4.79 eV, respectively. Considering the surface work function of HZO is 3.79 eV, there will be more holes transferred to HZO films grown on LSMO than those grown on LMO. Therefore, the *o*-HZO phase is more favorable on the MnO_2_-terminated LSMO than that on LMO.

In summary, we have demonstrated an enhanced ferroelectric *o*-phase in HZO thin films by engineering the termination of the HZO/LSMO interface. Such interface engineering permits stabilization of the metastable ferroelectric *o*-phase in epitaxial HZO thin films, which originates from hole doping from the MnO_2_-terminated LSMO to the HZO layer. The insights gained in this work establish a critical role of the LSMO termination layer in stabilizing the ferroelectric phase in HZO thin films and provide a clue for understanding ferroelectricity in hafnia-based films. This work also suggests a strategy to generate enhanced ferroelectric polarization in HZO thin films. We hope that our results will inspire further exploration of interface engineering to enhance ferroelectricity in hafnia-based systems.

## Methods

### Thin film deposition

Ferroelectric HZO films on La_0.67_Sr_0.33_MnO_3_ (LSMO) bottom electrodes were epitaxially grown on single-crystalline STO (001) substrates by pulsed laser deposition (PLD) using a KrF (λ = 248 nm) excimer laser. The bottom HZO/LSMO interface was fabricated either HZO/MnO_2_ or HZO/La_0.67_Sr_0.33_O terminated. LSMO thin films (20 u.c.) were grown at a substrate temperature of 950 °C with an oxygen pressure of 200 mTorr, while HZO films (1.5–8 nm) were deposited at a temperature of 800 °C with an oxygen pressure of 75 mTorr. The laser fluence was 1.25 J cm^−2^ with a repetition rate of 3 Hz. LSMO thin films were deposited with the layer-by-layer growth mode monitored by RHEED oscillations. In order to control the interface termination, STO substrates were treated with a buffered hydrofluoric acid etching process followed by thermal treatment at 875 °C for 3 h to get a TiO_2_-terminated surface before growth. The SrO-terminated surface was obtained by the growth of 1 u.c. of SrRuO_3_ (SRO) layer on top of treated STO substrates. The SRO thin layer was deposited at a temperature of 950 °C with an oxygen pressure of 5 mTorr and then left at 950 °C for 10 min. The laser fluence was 1.1 J cm^−2^ with a repetition rate of 1 Hz. At a high temperature, the RuO_2_ monolayer of SRO evaporated, leaving the SrO-terminated surface automatically. Control samples were fabricated using a 2-unit cell of LaMnO_3_ layer deposited between the HZO and LSMO layers to tune the charge state of the termination. Since LaMnO_3_ exhibits a MnO_2_-LaO-MnO_2_-LaO layer-by-layer growth mode during the PLD process, deposition of a 2-u.c. LaMnO_3_ at the interface results in either MnO_2_-termination with a negative charge of −1 or LaO-termination with a positive charge of +1. After the deposition, the films were cooled down to room temperature at an oxygen pressure of 1 torr with a cooling rate of 10 °C/min. The top Pt layer was deposited by magnetron sputtering without breaking the vacuum by transferring the sample from the PLD system into the sputtering system for in situ growth. Then, an array of 10 × 10 µm^2^ top Pt electrodes was patterned via photolithography and ion etching for electrical measurements.

### Ferroelectricity measurements

The ferroelectric measurements (P-E hysteresis loops) were performed using a Radiant Precision Multiferroic II tester. Ferroelectric polarization and local hysteresis loops of HZO thin films were characterized by piezoelectric force microscopy (PFM) using an Asylum Research MFP-3D instrument with Pt/Ti-coated tips. A 30 nm of Pt with a device feature of 10 × 10 µm^2^ was sputtered as the top electrode material prior to the ferroelectric test. For the out-of-plane P-E measurement, a bipolar triangular waveform was applied at frequencies ~100 kHz. For the in-plane P-E measurement, a bipolar triangular waveform was applied at frequencies ~20 kHz, with the interdigitated electrode spacing of around 5 µm.

### Material characterization

Microstructure of the samples, interfacial structure, EDX elemental mapping, and atomic layer-by-layer EELS were obtained by aberration-corrected STEM at room temperature. Cross-sectional TEM samples were prepared with a focused ion beam setup (DA300, FEI). The crystal structure and epitaxial quality were characterized by synchrotron XRD using a four-circle diffractometer with an X-ray wavelength of 1.5406 Å at the Singapore Synchrotron Light Source.

### Theoretical modeling and DFT calculations

Density functional theory (DFT) calculations were performed using the Vienna Ab-initio Simulation Package (VASP)^58^. Projector augmented-wave (PAW) potentials and the generalized gradient approximation (GGA) within the Perdew–Burke–Ernzerhof (PBE) parameterization were used to describe the electron-ion and the electronic exchange-correlation interactions^59,60^. The energy cut-off for the plane waves was set at 500 eV. The threshold for the energy convergence of the self-consistent loops was set at 10^−6^ eV. For structural optimization, the convergence of forces was set to be 10^−3^ eVÅ^−1^. The slab model was used to calculate the work functions of HZO, MnO_2_, LaO-, and LaSrO-terminated LSMO. We built the HZO slab from the orthorhombic ferroelectric bulk phase of HfO_2_, where half of the Hf atoms is substituted with Zr, along the nonpolar (100) direction, to eliminate the electric field in vacuum. Figure 4a shows the slab structure, which has the in-plane lattice of 5.073 Å × 5.098 Å, contains 8 Hf, 8 Zr, and 12 O atoms, and has a 15 Å vacuum layer to exclude interaction between the slab and its periodic images. For MnO_2_-, LaO-, and LaSrO-terminated LSMO models, to simplify the calculations, we assumed the LSMO stoichiometry to be represented by La_0.75_Sr_0.25_MnO_3_ and LaSrO-termination to be La_0.5_Sr_0.5_O. We cut the slabs from the ferromagnetic LaMnO_3_ bulk phase (space group of $P{{{{{\rm{nma}}}}}}$) along the (001) direction, and substituted a quarter of La atoms with Sr. The LSMO slabs of different terminations are shown in Fig. 4a. For all these slabs, in plan lattices of 5.526 Å × 5.544 Å and 15 Å vacuum layer are applied, and ferromagnetic order is utilized. Our calculations show that the work function difference between *o*-HZO and LSMO is relatively large. Therefore, even though the work function of La_0.67_Sr_0.33_MnO_3_ may be slightly different from that of La_0.75_Sr_0.25_MnO_3_, the main trend found here will not change. To simulate charge doping effects on the phase stability of HZO, an electronic charge of a certain density was added (electron doping) or removed (hole doping) to bulk HZO on a charge-neutralizing positive or negative background. Then the corresponding structures were fully optimized, and the total energies of *o*- and *m*-phases were calculated.

### Reporting summary

Further information on research design is available in the Nature Portfolio Reporting Summary linked to this article.

## Supplementary information

Supplementary Information

Reporting Summary

## Data Availability

The data that support the findings of this study are available from the corresponding author upon reasonable request. Source data are provided with this paper.

## Source data

Source Data
