# Supplementary material for: Interface-engineered ferroelectricity of epitaxial Hf0.5Zr0.5O2 thin films
Source: Nat Commun. 2023 Mar 30;14:1780. doi: 10.1038/s41467-023-37560-3 (PMC10063548; doi:10.1038/s41467-023-37560-3)
Supplement: Supplementary file 1 — Supplementary Information [file 41467_2023_37560_MOESM1_ESM.pdf]

Supplementary materials for

Interface-engineered ferroelectricity of epitaxial $Hf_{0.5}Zr_{0.5}O_2$ thin films

Shu Shi[1†], Haolong Xi[2,3†], Tengfei Cao[4†], Weinan Lin[5†], Zhongran Liu[3], Jiangzhen Niu[6], Da Lan[1], Chenghang Zhou[1], Jing Cao[7], Hanxin Su[1], Tieyang Zhao[1], Ping Yang[8], Yao Zhu[9], Xiaobing Yan[6*], Evgeny Y. Tsymbal[4*], He Tian[3,10*], Jingsheng Chen[1*]

[1]Department of Materials Science and Engineering, National University of Singapore, 117575 Singapore, Singapore

[2]School of Materials and Energy, Electron Microscopy Centre of Lanzhou University and Key Laboratory of Magnetism and Magnetic Materials of the Ministry of Education, Lanzhou University, Lanzhou 730000, PR China

[3]Center of Electron Microscope, State Key Laboratory of Silicon Materials, School of Materials Science and Engineering, Zhejiang University, Hangzhou 310027, China

[4]Department of Physics and Astronomy and Nebraska Center for Materials and Nanoscience, University of Nebraska, Lincoln, NE 68588-0299, USA

[5]Department of physics, Xiamen University, Xiamen 361005, China

[6]Key Laboratory of Brain-Like Neuromorphic Devices and Systems of Hebei Province, Hebei University, Baoding 071002, PR China

*[7]Institute of Materials Research and Engineering, Agency for Science, Technology and Research (A*STAR), 138634 Singapore, Singapore*

[8]Singapore Synchrotron Light Source (SSLS), National University of Singapore, 5 Research Link, 117603 Singapore, Singapore

*[9]Institute of Microelectronics, Agency for Science, Technology and Research (A*STAR), 138634 Singapore, Singapore*

[10]School of Physics and Microelectronics, Zhengzhou University, Zhengzhou, 450052, China

†These authors contributed equally to this work.

*email: msecj@nus.edu.sg (J.C.), hetian@zju.edu.cn (H.T.), tsymbal@unl.edu (E.Y.T.), yanxiaobing@ime.ac.cn (X.Y.)

1. Thickness of the HZO layer

The thickness of HZO films was measured by x-ray reflectivity (XRR), as shown in Fig. S1. The clear oscillations resulted from the HZO and LSMO layer indicate the smooth interface and surface.

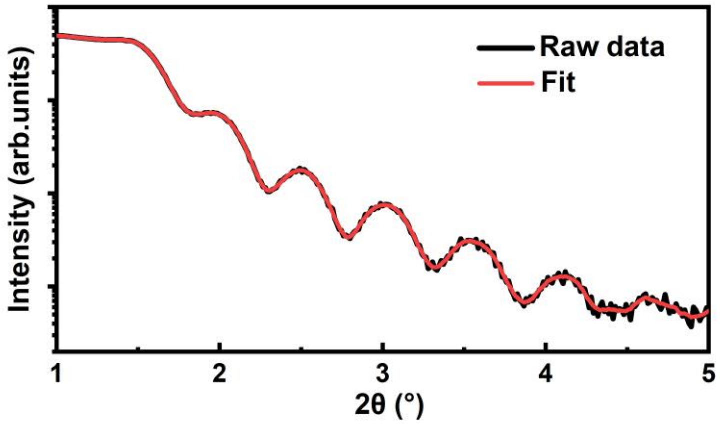

Supplementary Figure S1 | X-ray reflectivity (XRR) of the HZO/LSMO/STO (001) thin film. The black and red curves correspond to the raw XRR data and the fitted data, respectively.

2. Enhanced fraction of ferroelectric *o*-phase by interfacial engineering

The XRD ω-2θ scans for HZO films with the film thickness of 1.5-8 nm are displayed in Fig. S2. As the thickness reduces, the characteristic peak of HZO shifts to left, with the width of peak becoming more broad due to the thickness effect. It is observed that, being independent of the HZO thickness, the A-type heterostructure exhibits a stronger *o*-phase peak and a weaker *m*-phase peak compared to the B-type heterostructure. Notably, the clear ferroelectric *o*-phase (111) crystal orientation peak is observed on the A-type heterostructure HZO film even though the HZO thickness is as thin as 1.5 nm, while no such peak can be observed on the B-type heterostructure HZO film. This result indicates that our interfacial engineering strategy can push the fundamental limits of the ferroelectric HZO *o*-phase to below 1.5 nm.

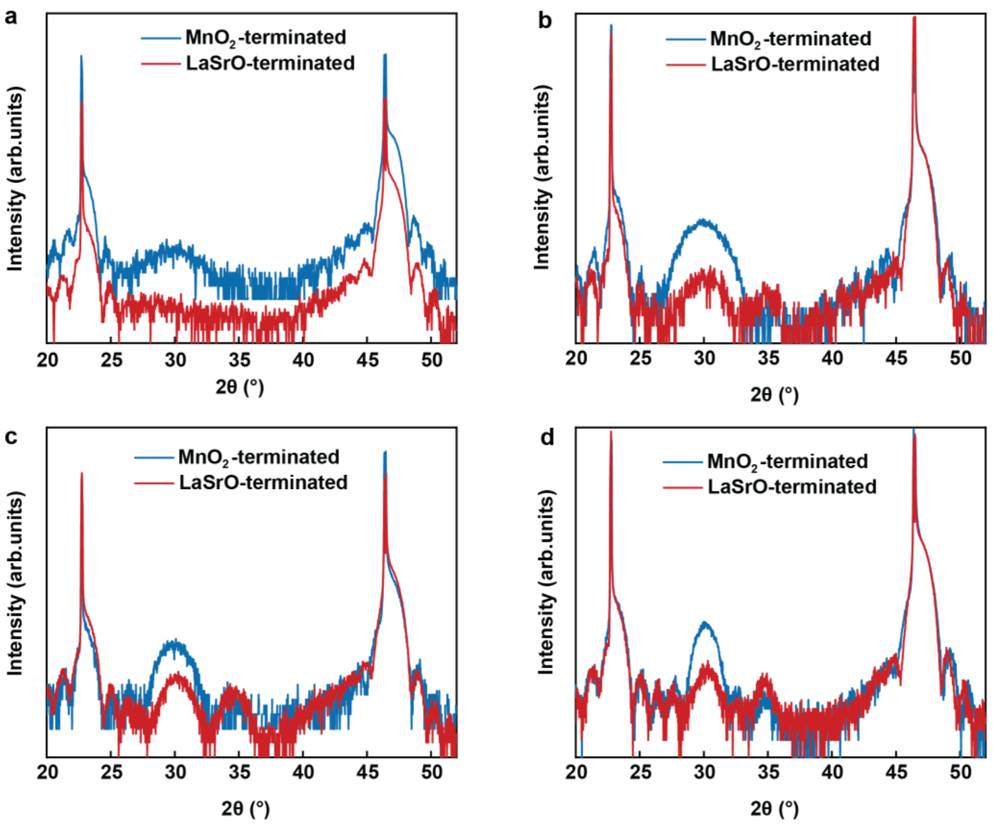

Supplementary Figure S2 | XRD data for A-type and B-type heterostructures with different HZO layer thickness (t_{HZO}). a, t_{HZO} = 1.5 nm, **b,** t_{HZO} = 3 nm, **c,** t_{HZO} = 5 nm, **d,** t_{HZO} = 8nm.

3. In-plane P-E loop measurement of ultrathin HZO film

The in-plane P-E loop measurement is performed for ultrathin HZO film (1.5 nm) with an A-type heterostructure to overcome its leakage challenges, as shown in Fig. S3. It is worth noting that for 1.5 nm HZO film with the B-type heterostructure, no typical ferroelectric P-E loop can be obtained, indicating the non-ferroelectric characteristic of the sample. This result is in line with the XRD data (Fig. 1e) that no ferroelectric *o*-phase is observed in the B-type heterostructure with HZO film thickness of 1.5 nm.

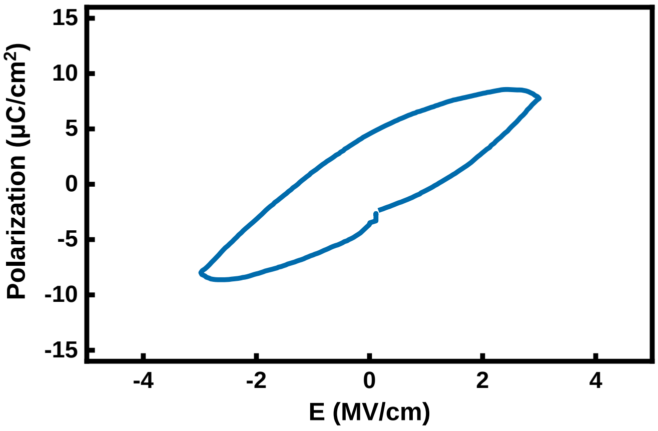

Supplementary Figure S3 | In-plane polarization-electric field (P-E) loop for the A-type heterostructure. The HZO layer thickness is t_{HZO} = 1.5 nm.

4. Typical ferroelectric current-electric field (I-E) measurement of HZO films

Ferroelectric I-E curves for HZO films with the A- and B-type heterostructure are shown in Fig. S4, in response to the application of a voltage sweep at 100 kHz to the pristine sample with a ~ 5 MV/cm amplitude. The ferroelectric switching current is clearly shown with coercive field, E_c, around 4.6 MV/cm and 4.5 MV/cm for A- and B-type heterostructure HZO films, respectively.

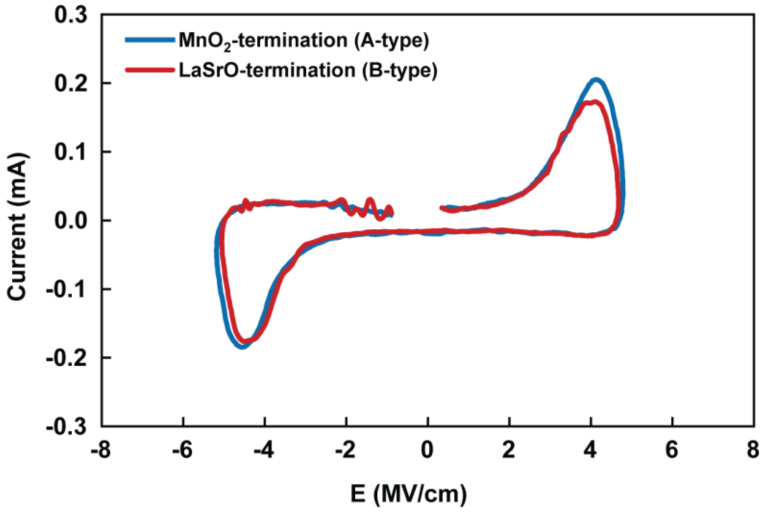

Supplementary Figure S4 | Typical ferroelectric current-electric field (I-E) switching curves. I-E curves for A-type (blue-curve) and B-type (red-curve) heterostructures. HZO layer thickness is t_{HZO} = 8 nm.

5. Wake-up effect and endurance of HZO films

Figure S5a, b present the wake up test for the HZO film with two types of heterostructures. In A-type heterostructure, a minimal increase of the polarization after 10 cycles compared with the pristine state is observed. Also notice that the P_r in the 10th cycle is almost the same as P_r in the 10^9 cycle, which indicates no wake-up in this sample. Note that for B-type heterostructure, there is an obvious increase of P_r in the first 10000 cycles, suggesting wake-up behavior. The polarization window ($2P_r$) is plotted against the number of cycles of two types of heterostructures in Fig. S5c, d. The HZO film shows good endurance without a breakdown after 10^9 cycles.

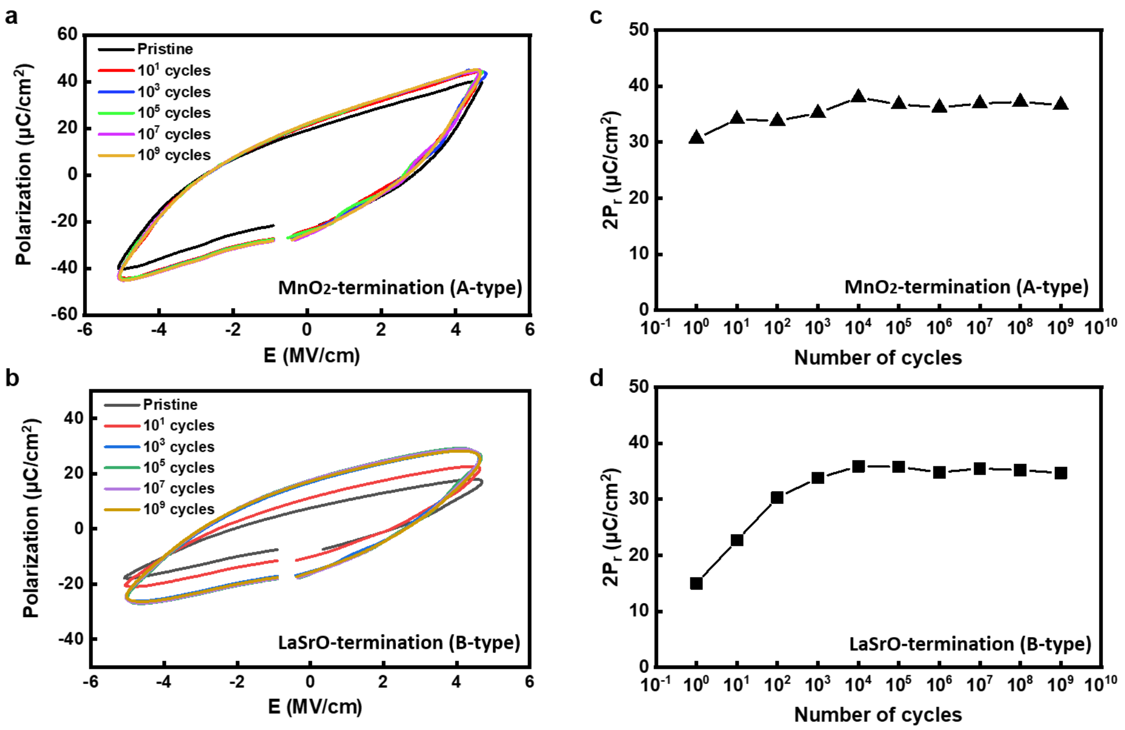

Supplementary Figure S5 | Wake up and endurance tests for the HZO/LSMO/STO (001) thin film of two heterostructures. a, b, Polarization-electric field loops with the number of cycles for A- and B-type heterostructures. **c, d,** Polarization window ($2P_r$) as a function of the number of cycles of A- and B-type heterostructures. The HZO layer thickness is t_{HZO} = 8 nm.

6. Ferroelectric switching behavior of HZO films

The ferroelectric behavior of the two types of heterostructures is investigated by piezo-response force microscopy (PFM) technique and ferroelectric testing system. Firstly, we performed electrical poling measurements to characterize the ferroelectric domain switching using ±7 V poling voltage. Figure S6a, b show the out-of-plane PFM phase images measured for the A- and B-type samples. It shows that applying a bias voltage of +7 V or -7 V, the virgin state of HZO changes to a state with polarization pointing down (indicated by the yellow contrast in Fig. S6a, b) or up (indicated by the purple contrast), respectively. The polarization is reversed when an opposite bias is applied. Next, we performed switching spectroscopy PFM (SS-PFM) to investigate the hysteretic behavior of the two heterostructures. Figure S6c, d show a typical local PFM phase loop and butterfly-like amplitude loop of HZO thin films. These results demonstrate a standard local hysteretic electromechanical response, indicating switchable polarization of the HZO films in both types of structures.

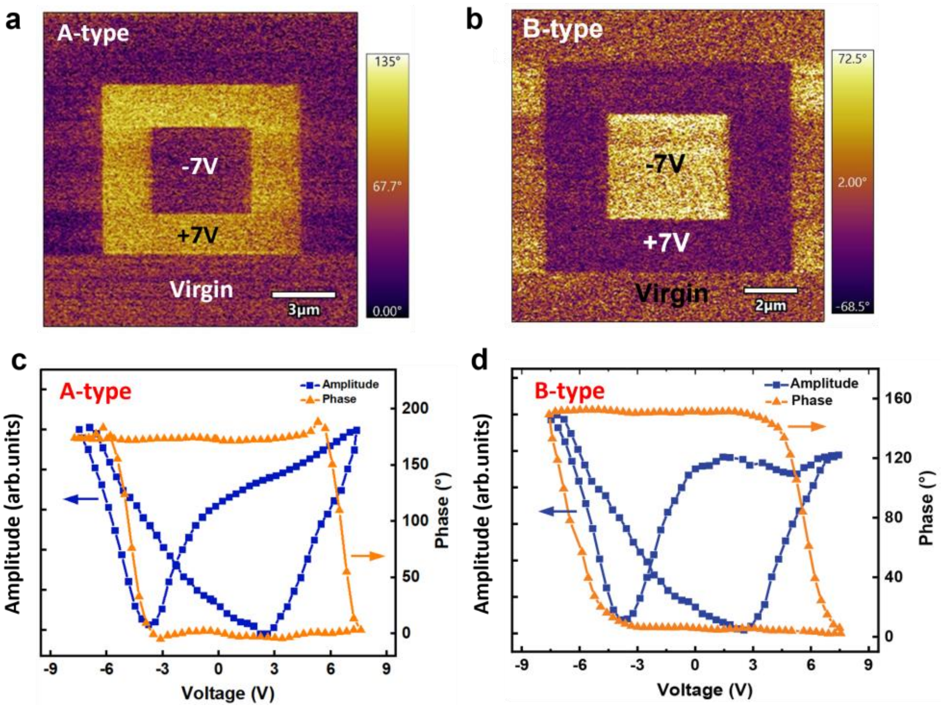

Supplementary Figure S6 | PFM measurement of A- and B-type heterostructures. a, b, Out of plane PFM images of A- and B-type heterostructures measured on HZO films after the film deposition. The yellow and purple contrasts in the PFM phase images represent the upward and downward polarization direction, respectively. **c, d,** Single point hysteresis loop for A- and B-type heterostructures obtained using SS-PFM method. Blue square represents amplitude change and orange triangle represents phase change.

7. Topography and retention of the PFM contrast of HZO films

The corresponding topography and amplitude of the HZO films before and after poling for HZO films with film thickness of 8 nm (Fig. S7) and 1.5 nm (Fig. S8) are shown to rule out artifacts from charge injection and electrochemical origins. Fig. S7a, b and Fig. S7e, f show the PFM contrast for A-type and B-type heterostructures with HZO film thickness of 8 nm at 0 min and at 60 min, respectively. Fig. S8a, e show the PFM contrast for A-type heterostructure with HZO film thickness of 1.5 nm at 0 min and 60 min, respectively. The result shows good retention of ferroelectric switching of both 8 nm and 1.5 nm HZO films. For the B-type heterostructure with HZO film thickness of 1.5 nm, a clear ferroelectric switching PFM contrast is very difficult to be obtained (Fig. S8c). This result is in line with the XRD data (Fig. 1e) that no ferroelectric *o*-phase is observed in the B-type heterostructure with HZO film thickness of 1.5 nm.

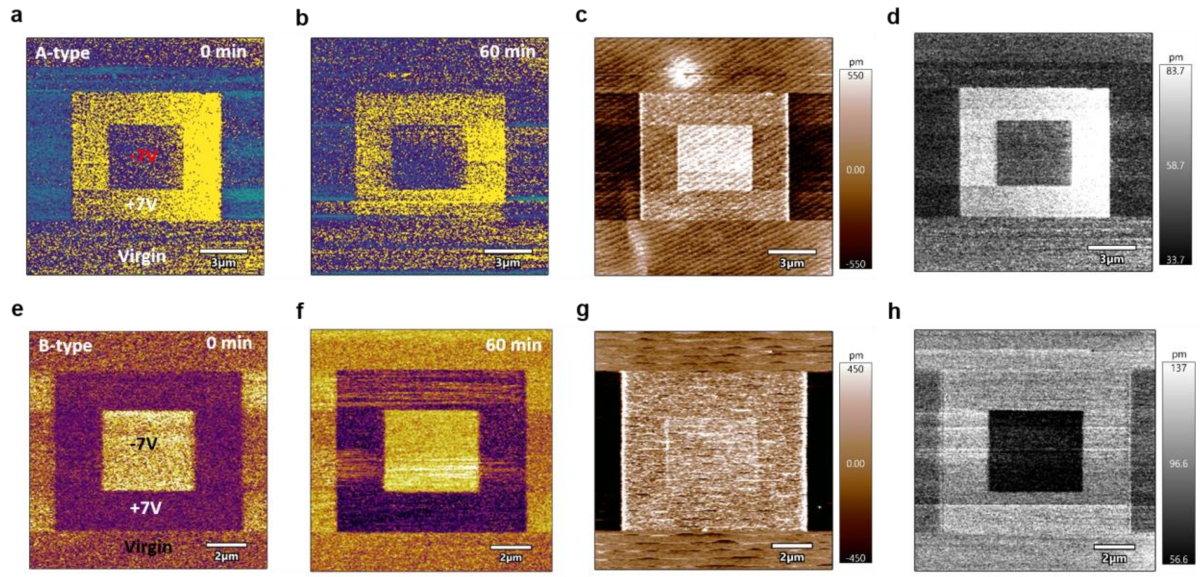

Supplementary Figure S7 | PFM measurement of A- and B-type heterostructures with t_{HZO} = 8 nm. **a, b**, Out of plane PFM images of A-type heterostructure measured at 0 min and 60 min, respectively. The yellow and blue-purple contrasts in the PFM phase images represent the upward and downward polarization direction, respectively. **c, d**, The corresponding topography and amplitude of A-type heterostructure. **e, f**, Out of plane PFM images of B-type heterostructure measured at 0 min and 60 min, respectively. The yellow and purple contrasts in the PFM phase images represent the upward and downward polarization direction, respectively. **g, h**, The corresponding topography and amplitude of B-type heterostructure.

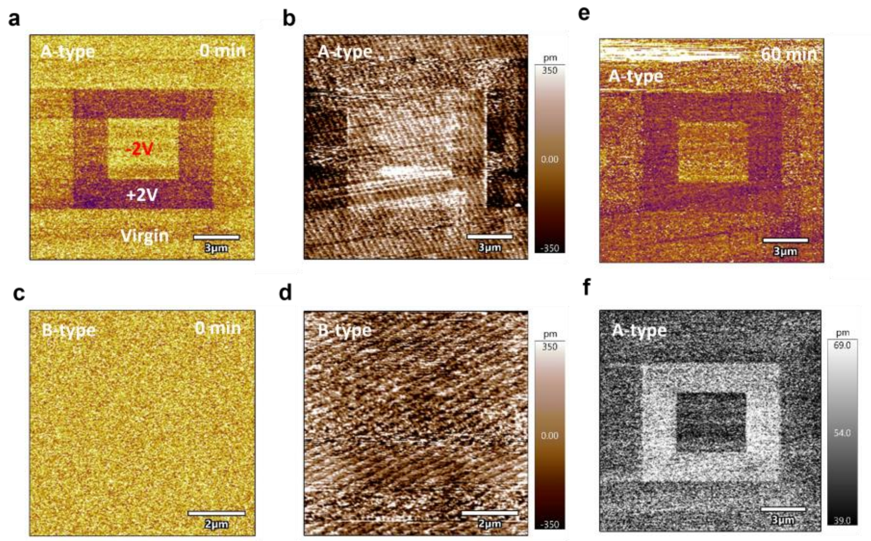

Supplementary Figure S8 | PFM measurement of A-type and B-type heterostructures with t_{HZO} = 1.5 nm. **a, c**, Out of plane PFM images of A- and B-type heterostructure measured at 0 min, respectively. The yellow and purple contrasts in the PFM phase images represent the upward and downward polarization direction, respectively. **b, d**, The corresponding topography of A- and B-type heterostructure, respectively. **e**, Out of plane PFM image of A-type heterostructure measured at 60 min. **f**, The corresponding amplitude of A-type heterostructure.

8. Phase identification

The *o*-phase and *m*-phase identification is confirmed by analyzing the atomic structure in HAADF-STEM images in combining with the Fast Fourier Transform (FFT). By comparing HAADF-STEM with the corresponding standard atomic structure model and FFT calibration, the *o*-phase [110], [101], [010] orientation and *m*-phase [110] orientation in the samples were verified (Fig. S9a-d). In addition, multiple groups of crystal planar spacing corresponding to *o*-phase and *m*-phase are measured, respectively. The results showed that there was almost no difference between the spacing of crystal planes corresponding to their standard structures (Fig. S9e, f), which strongly supports our analysis. The way chosen to distinguish *o*-phase and *m*-phase is common and well-accepted, which is widely conducted in the community, such as reports in references [1-3].

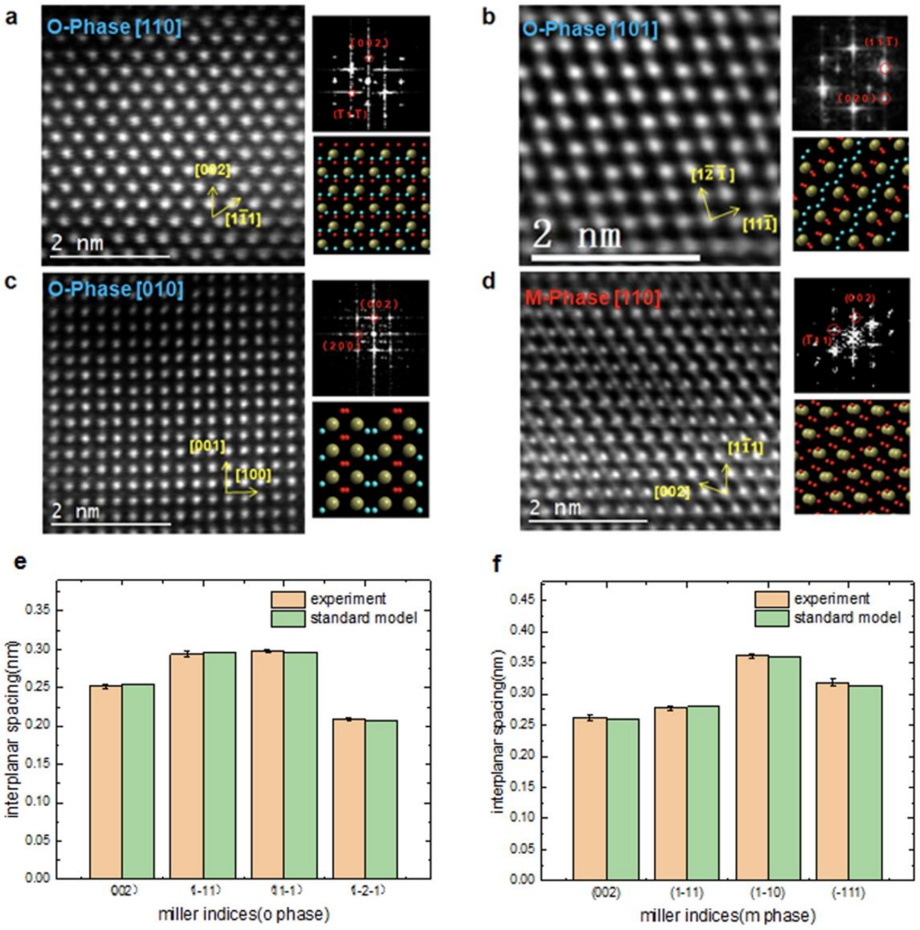

Supplementary Figure S9 | Structure and phase analysis. **a-d**, Atomic-scale HAADF STEM images, corresponding fast Fourier transform (FFT) and structure model of *o*-phase and *m*-phase orientation. Hf atom is represented by yellow (large), and O atom is represented by red and cyan (small). **e-f**, The experiment (orange bars) and standard (green bars) model interplanar spacing of *o*-phase and *m*-phase, respectively. Error bars are calculated from the interplane spacing measurements of different regions for each phase.

9. Ferroelectricity in MnO_2-terminated ultrathin HZO film

Fig. S10 shows the STEM characterization and the combined FFT for the 1.5 nm HZO sample with the A-type heterostructure. The HZO demonstrates a highly textured growth on the bottom LSMO layer. At the same time, it is observed that HZO demonstrates *o*-phase, which is consistent with our macroscopic XRD result (Fig. 1e) of the A-type heterostructure.

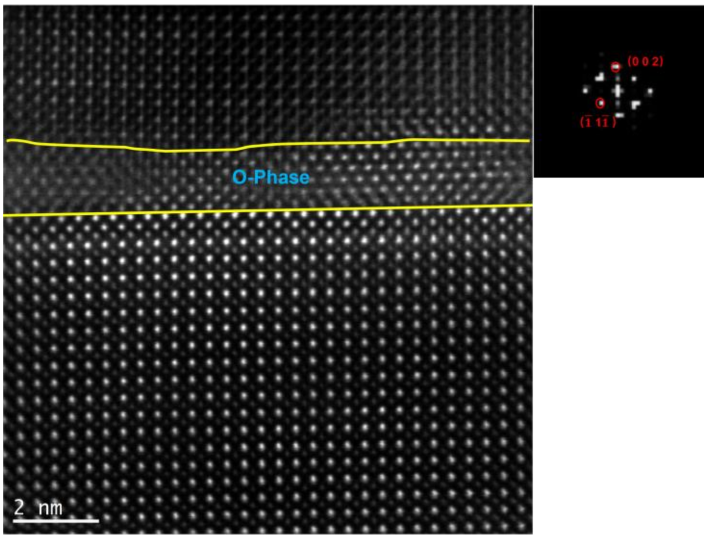

Supplementary Figure S10 | HAADF-STEM image and corresponding fast Fourier transform (FFT) of the A-type heterostructure with t_{HZO} = 1.5 nm.

10. Statistics of *m*-phase and *o*-phase crystalline grains in two heterostructures

Fig. S11a, b show a wide range of STEM images for both A-type heterostructure and B-type heterostructure. More than 50 regions of A-type and B-type heterostructure are observed to quantify the *o*-phase and the *m*-phase distribution in two heterostructures, respectively. Statistics on *o*-phase and *m*-phase of HZO grains are shown in Fig. S11c, d. In A-type heterostructure, the *o*-phase, *m*-phase and *t*-phase grain accounts for 76%, 22% and 2%, respectively. In B-type heterostructure, *o*-phase grains account for only 32%, *m*-phase grains account for 64% and *t*-phase grains account for 4%. The statistical measurement results show that A-type (B-type) heterostructure has more *o*-phase (*m*-phase).

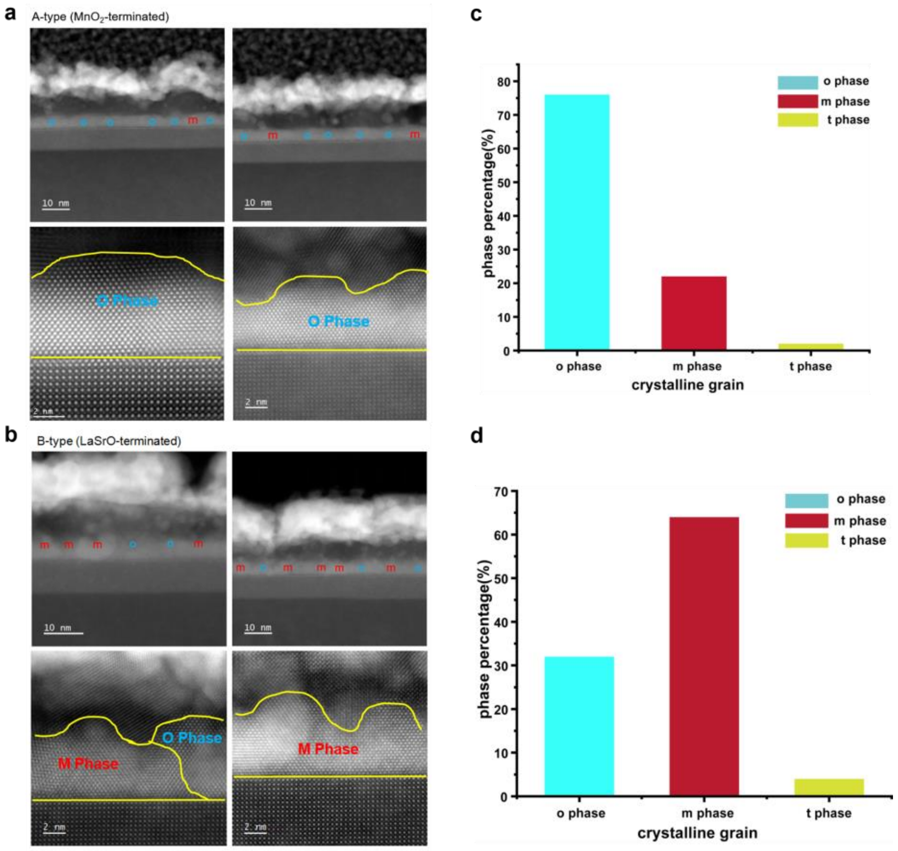

Supplementary Figure S11 | Statistics of *m*-phase and *o*-phase crystalline grains. **a, b**, Low and high magnification HAADF-STEM of A- and B-type heterostructure. The *o*-phase (blue) and *m*-phase (red) of low magnification HAADF-STEM are marked. **c, d**, Statistics percentage

of *o*-phase, *m*-phase and *t*-phase crystalline grains in A- and B-type heterostructure, respectively.

Reference:

1. Kang S, *et al.* Highly enhanced ferroelectricity in HfO_2-based ferroelectric thin film by light ion bombardment. *Science* **376**, 731-738 (2022).

2. Xu X, *et al.* Kinetically stabilized ferroelectricity in bulk single-crystalline HfO(2):Y. *Nat Mater* **20**, 826-832 (2021).

3. Zhong H, *et al.* Large-Scale $Hf_{0.5}Zr_{0.5}O_2$ Membranes with Robust Ferroelectricity. *Adv Mater*, 2109889 (2022).
