# Supplementary material for: Interface-engineered ferroelectricity of epitaxial Hf0.5Zr0.5O2 thin films
Source: Nat Commun. 2023 Mar 30;14:1780. doi: 10.1038/s41467-023-37560-3 (PMC10063548; doi:10.1038/s41467-023-37560-3)
Supplement: Supplementary file 2 — Reporting Summary [file 41467_2023_37560_MOESM2_ESM.pdf]

natureresearch

Corresponding author(s): Jingsheng Chen, He Tian, Evgeny Y. Tsymbal and Xiaobing Yan

Lasing Reporting Summary

Nature Research wishes to improve the reproducibility of the work that we publish. This form is intended for publication with all accepted papers reporting claims of lasing and provides structure for consistency and transparency in reporting. Some list items might not apply to an individual manuscript, but all fields must be completed for clarity.

For further information on Nature Research policies, including our data availability policy, see Authors & Referees.

ü Experimental design

Please check: are the following details reported in the manuscript?

1. Threshold

Plots of device output power versus pump power over a wide range of values indicating a clear threshold

- [ ] Yes
- [x] No

Since our work demonstrates the properties of the thin films fabricated by Pulsed Laser Deposition technique rather than a new report of lasing per se, we did not feel it necessary to provide the typical "laser graph" showing output power vs pump from well below threshold.

2. Linewidth narrowing

Plots of spectral power density for the emission at pump powers below, around, and above the lasing threshold, indicating a clear linewidth narrowing at threshold

- [ ] Yes
- [x] No

Since our work demonstrates the properties of the thin films fabricated by Pulsed Laser Deposition technique rather than a new report of lasing per se, these data are not directly related to our study.

Resolution of the spectrometer used to make spectral measurements

- [ ] Yes
- [x] No

Since our work demonstrates the properties of the thin films fabricated by Pulsed Laser Deposition technique rather than a new report of lasing per se, these data are not directly related to our study.

3. Coherent emission

Measurements of the coherence and/or polarization of the emission

- [ ] Yes
- [x] No

Since our work demonstrates the properties of the thin films fabricated by Pulsed Laser Deposition technique rather than a new report of lasing per se, these data are not directly related to our study.

4. Beam spatial profile

Image and/or measurement of the spatial shape and profile of the emission, showing a well-defined beam above threshold

- [ ] Yes
- [x] No

Since our work demonstrates the properties of the thin films fabricated by Pulsed Laser Deposition technique rather than a new report of lasing per se, these data are not directly related to our study.

5. Operating conditions

Description of the laser and pumping conditions
Continuous-wave, pulsed, temperature of operation

- [x] Yes
- [ ] No

Page 16, "Methods-Thin film deposition" section

Threshold values provided as density values (e.g. W cm^{-2} or J cm^{-2}) taking into account the area of the device

- [ ] Yes
- [x] No

We did not specify the threshold as a density value as this is not directly related to our study. However we do give the energy density of the laser fluence in our experiment.

6. Alternative explanations

Reasoning as to why alternative explanations have been ruled out as responsible for the emission characteristics
e.g. amplified spontaneous, directional scattering; modification of fluorescence spectrum by the cavity

- [ ] Yes
- [x] No

Since our work demonstrates the properties of the thin films fabricated by Pulsed Laser Deposition technique rather than a new report of lasing per se, this is not a necessary consideration.

7. Theoretical analysis

Theoretical analysis that ensures that the experimental values measured are realistic and reasonable
e.g. laser threshold, linewidth, cavity gain-loss, efficiency

- [ ] Yes
- [x] No

Since our work demonstrates the properties of the thin films fabricated by Pulsed Laser Deposition technique rather than a new report of lasing per se, this is not a necessary consideration.

8. Statistics

Number of devices fabricated and tested

- [ ] Yes
- [x] No

Since our work demonstrates the properties of the thin films fabricated by Pulsed Laser Deposition technique rather than a new report of lasing per se, this is not a necessary consideration.

Statistical analysis of the device performance and lifetime (time to failure)

☐ Yes
☒ No

There was no statistical analysis on this device.
